# Effect of *Porphyromonas gingivalis* infection on post-transcriptional regulation of the low-density lipoprotein receptor in mice

**DOI:** 10.1186/1476-511X-11-121

**Published:** 2012-09-19

**Authors:** Haruna Miyazawa, Koichi Tabeta, Sayuri Miyauchi, Yukari Aoki-Nonaka, Hisanori Domon, Tomoyuki Honda, Takako Nakajima, Kazuhisa Yamazaki

**Affiliations:** 1 Center for Transdisciplinary Research, Niigata University, 5274 Gakkocho 2-ban-cho, Chu-o-ku, Niigata, 951-8514, Japan; 2 Laboratory of Periodontology and Immunology, Division of Oral Science for Health Promotion, Niigata University Graduate School of Medical and Dental Sciences, 5274 Gakkocho 2-ban-cho, Chu-o-ku, Niigata, 951-8514, Japan; 3 General Dentistry and Clinical Education Unit, Niigata University Medical and Dental Hospital, 5274 Gakkocho 2-ban-cho, Chu-o-ku, Niigata, 951-8514, Japan

**Keywords:** PCSK9, LDL cholesterol, Periodontitis, Chronic inflammation

## Abstract

**Background:**

Periodontal disease is suggested to increase the risk of atherothrombotic disease by inducing dyslipidemia. Recently, we demonstrated that proprotein convertase subtilisin/kexin type 9 (PCSK9), which is known to play a critical role in the regulation of circulating low-density lipoprotein (LDL) cholesterol levels, is elevated in periodontitis patients. However, the underlying mechanisms of elevation of PCSK9 in periodontitis patients are largely unknown. Here, we explored whether *Porphyromonas gingivalis,* a representative periodontopathic bacterium, -induced inflammatory response regulates serum PCSK9 and cholesterol levels using animal models.

**Methods:**

We infected C57BL/6 mice intraperitoneally with *Porphyromonas gingivalis*, a representative strain of periodontopathic bacteria, and evaluated serum PCSK9 levels and the serum lipid profile. PCSK9 and LDL receptor (LDLR) gene and protein expression, as well as liver X receptors (*Lxrs*), inducible degrader of the LDLR (*Idol*), and sterol regulatory element binding transcription factor (*Srebf*)*2* gene expression, were examined in the liver.

**Results:**

*P. gingivalis* infection induced a significant elevation of serum PCSK9 levels and a concomitant elevation of total and LDL cholesterol compared with sham-infected mice. The LDL cholesterol levels were significantly correlated with PCSK9 levels. Expression of the *Pcsk9*, *Ldlr*, and *Srebf2* genes was upregulated in the livers of the *P. gingivalis*-infected mice compared with the sham-infected mice. Although *Pcsk9* gene expression is known to be positively regulated by sterol regulatory element binding protein (SREBP)2 (human homologue of Srebf2), whereas *Srebf2* is negatively regulated by cholesterol, the elevated expression of *Srebf2* found in the infected mice is thought to be mediated by *P. gingivalis* infection.

**Conclusions:**

*P. gingivalis* infection upregulates PCSK9 production via upregulation of *Srebf2*, independent of cholesterol levels. Further studies are required to elucidate how infection regulates *Srebf2* expression and subsequently influences lipid metabolism.

## Background

Periodontitis is associated with atherosclerotic vascular disease, as it induces dyslipidemia. It has been found that periodontitis decreases HDL cholesterol and increases LDL cholesterol levels. It has also been reported that the presence of periodontal pockets was positively associated with higher LDL and total cholesterol in humans [1]. Löesche *et al*. showed that the levels of total and LDL cholesterol were significantly higher in 50- to 60-year-old patients with moderate periodontitis compared with age- and sex-matched controls [2]. Furthermore, intensive periodontal therapy consisting of subgingival mechanical debridement with adjunctive local delivery of minocycline significantly decreased total and LDL cholesterol compared with baseline levels [3]. The mechanisms underlying the elevation of LDL cholesterol levels in periodontitis patients have not yet been elucidated.

Plasma cholesterol levels are regulated by the LDL receptor (LDLR). The number of LDLRs expressed on the hepatocyte surface is the primary determinant of plasma cholesterol levels and is therefore strictly regulated. Transcription of the LDLR gene is controlled by cellular cholesterol levels through the sterol regulatory element-binding protein (SREBP) [4] and Liver X receptors (LXRs) [5]. Additionally, post-transcriptional regulation of LDLR expression is a major determinant of lipoprotein metabolism.

Proprotein convertase subtilisin/kexin type 9 (PCSK9) post-transcriptionally promotes the degradation of LDLRs in hepatocytes [6]. PCSK9 is mainly expressed in the liver, intestine, and kidney, and it is secreted into the plasma [7]. This protein does not directly degrade LDLRs but binds to LDLRs at epidermal growth factor-like repeats. This binding decreases LDLR recycling to the cell surface and promotes lysosomal degradation [8], which results in decreased numbers of LDLRs and increased plasma LDL levels. We have recently shown that serum levels of PCSK9 are significantly increased in periodontitis patients compared with periodontally healthy subjects [9]. In addition to PCSK9, the inducible degrader of the LDL receptor (Idol) has been shown to play an important role in the post-transcriptional regulation of LDLRs. Zelcer *et al*. [5] found that LDLR expression is controlled by the LXR-Idol axis as LXR induces Idol, which is an E3 ubiquitin ligase that triggers LDLR degradation.

It is well-recognized that infection and inflammation induce marked changes in lipid and lipoprotein metabolism [10]. However, there is little information about the effects of infection on PCSK9 or Idol levels and subsequent expression of LDLR and cholesterol levels. Only one study has shown that inflammatory stimuli, such as lipopolysaccharide or zymosan, markedly increased hepatic PCSK9 mRNA expression, resulting in decreased LDLR protein expression [11]. Therefore, in the present study, we analyzed the effects of infection with *Porphyromonas gingivalis*, a representative strain of periodontopathic bacteria, on the regulation of PCSK9 and subsequently expression of LDLR in the liver.

## Results

### Serum SAA and PCSK9 levels

**Figure 1:**
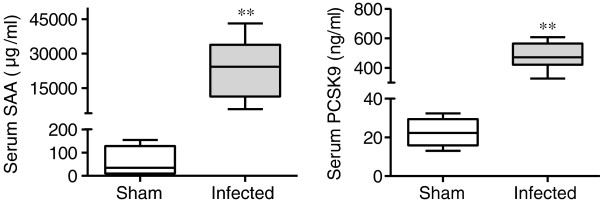
**Effects of *P. gingivalis *infection on serum levels of serum amyloid A (SAA) and PCSK9 (N = 5 in the sham-infected group; N = 6 in the infected group).** All samples were analyzed in duplicate for each condition. Box plots represent medians, with the 25^th^ and 75^th^ percentiles shown as boxes and the 10^th^ and 90^th^ percentiles as whiskers. Significant differences were observed between the infected group and the sham-infected group (** *P* < 0.01, Mann–Whitney U-test).

Systemic infection with *P. gingivalis* induced a significant elevation of serum amyloid A (SAA) levels. The PCSK9 levels in the infected mice were significantly higher than in the sham-infected mice (Figure 1).

### Plasma lipid profiles

**Figure 2:**
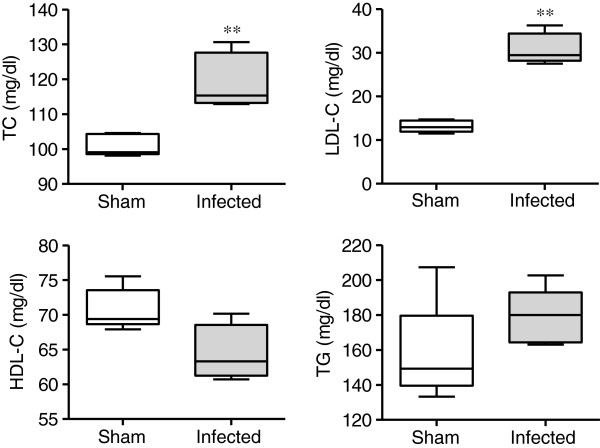
**Effects of *P. gingivalis *infection on the plasma lipid profile (N = 5 in each group).** Box plots represent medians, with the 25^th^ and 75^th^ percentiles as boxes and the 10^th^ and 90^th^ percentiles as whiskers. Total and LDL cholesterol levels were significantly higher, whereas HDL cholesterol levels tended to be lower in the *P. gingivalis*-infected mice (** *P* < 0.01, Mann–Whitney U-test).

The total and LDL cholesterol levels were significantly higher in the *P. gingivalis*-infected mice compared with the sham-infected mice. In contrast, HDL cholesterol levels tended to be lower in the *P. gingivalis*-infected mice than the sham-infected mice. There was no difference in triglyceride levels between the *P. gingivalis*-infected mice and sham-infected mice (Figure 2).

### Relationship between serum PCSK9 and LDL cholesterol levels

**Figure 3:**
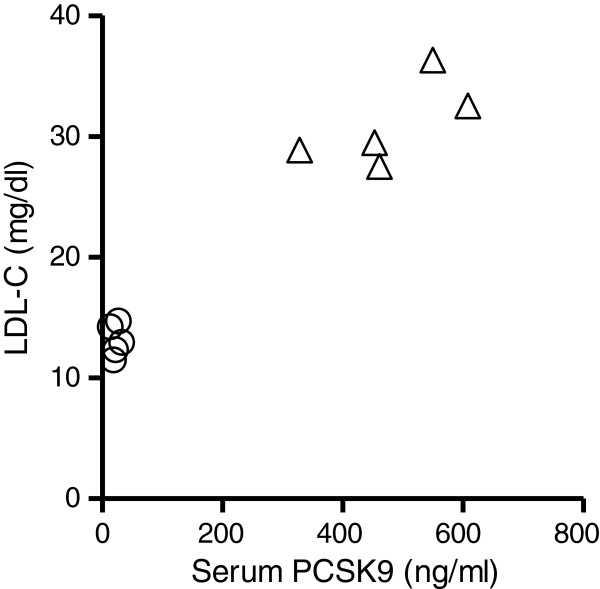
**Relationship between serum PCSK9 and LDL cholesterol levels in the *P. gingivalis*-infected (open triangle) and sham-infected (open circle) mice (N = 5 in each group).** Spearman’s rank correlation coefficient analysis showed that there was a significant correlation between LDL cholesterol levels and serum PCSK9 levels. Spearman r = 0.8545, *P* < 0.01.

Similar to a previous study by our group that demonstrated a close relationship between PCSK9 and LDL cholesterol, we found a significant correlation between PCSK9 levels and LDL cholesterol levels after *P. gingivalis* infection (Figure 3).

However, no correlations were observed between PCSK9 levels and total cholesterol levels, HDL cholesterol levels, or triglyceride levels (data not shown).

### PCSK9 and LDLR gene and protein expression in the liver

**Figure 4:**
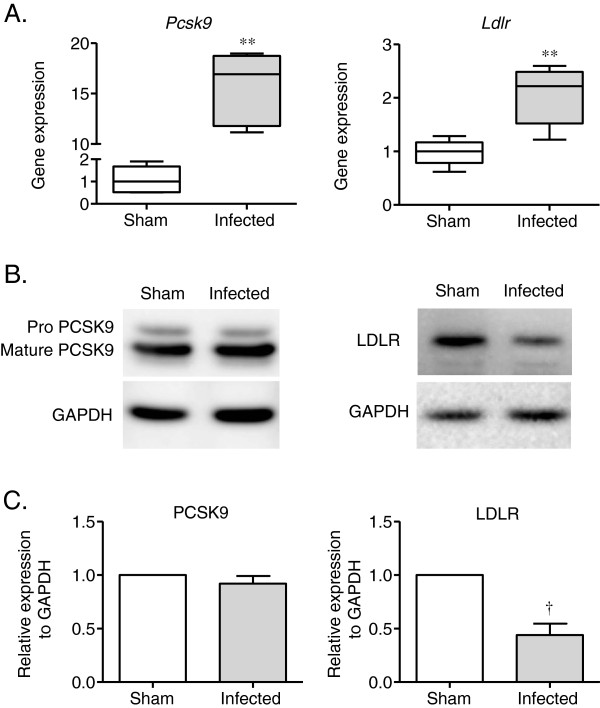
**Effect of *P. gingivalis *infection on PCSK9 and LDLR gene and protein expression in the liver.** Total RNA and protein were extracted from the *P. gingivalis*-infected and sham-infected mice, and the samples were analyzed by quantitative RT-PCR or western blotting. *Pcsk9* and *Ldlr* gene expression was significantly higher in the *P. gingivalis*-infected mice compared with the sham-infected mice (** *P* < 0.01, Mann–Whitney U-test). The PCSK9 protein levels of were unchanged in the *P. gingivalis*-infected mice compared with the sham-infected mice. LDLR protein levels were significantly lower in the *P. gingivalis*-infected mice compared with the sham-infected mice (^†^*P* < 0.05, unpaired t-test).

There was a significant increase in the expression of PCSK9 and LDLR (Figure 4A) in the infected mice compared to the sham-infected mice. However, there was a much larger increase in gene expression observed for PCSK9 than LDLR. Although *Pcsk9* gene expression was increased, the levels of mature PCSK9 protein in the liver of the *P. gingivalis*-infected mice were similar to those of the sham-infected mice. Pro-PCSK9 protein levels were even lower in the *P. gingivalis*-infected mice compared with the sham-infected mice. In contrast to the gene expression results, the protein levels of LDLR were significantly lower in the livers of the *P. gingivalis*-infected mice compared with the sham-infected mice (Figure 4B and C).

### Changes in *Srebf2*, *Lxrs*, and *Idol* gene expression

**Figure 5:**
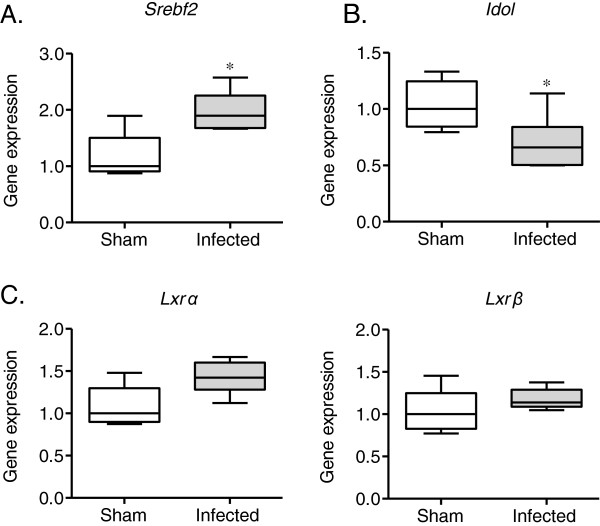
**Comparison of the relative gene expression levels in the liver between the *P. gingivalis*-infected mice and the sham-infected mice (N = 5 in the sham-infected group, N = 6 in the infected group).** The relative mRNA levels for the investigated genes were normalized to the relative glyceraldehyde-3-phosphate dehydrogenase (GAPDH) mRNA levels. The box plots represent medians, with the 25^th^ and 75^th^ percentiles being depicted as boxes and the 10^th^ and 90^th^ percentiles presented as whiskers. Significant differences were observed for *Srebf2* and *Idol* (* *P* < 0.05, Mann–Whitney U-test).

SREBP2 (Srebf2 in mice) is a major activator of PCSK9 signaling. Therefore, the effect of *P. gingivalis* infection on PCSK9 expression was analyzed. As shown in Figure 5A, *Srebf2* expression was significantly upregulated in the *P. gingivalis*-infected mice compared with the sham-infected mice.

Because of the important role of Idol in the post-transcriptional regulation of LDLR expression, the effects of *P. gingivalis* infection on *Idol* gene expression and the gene expression of *Lxrs*, a regulator of the *Idol* gene, were examined. *Idol* gene expression was slightly, but significantly suppressed in the *P. gingivalis*-infected mice compared with the sham-infected mice (Figure 5B). However, no differences in *Lxrα* or *Lxrβ* gene expression were observed, although the expression of both genes tended to be higher in the *P. gingivalis*-infected mice (Figure 5C).

## Discussion

Given that humans with loss-of-function mutations in the *PCSK9* gene [12] and mice lacking *Pcsk9* expression exhibit significantly reduced circulating LDL cholesterol levels [13], whereas activating mutations in the *PCSK9* gene result in severe familial hypercholesterolemia [14] accompanied by increased cardiovascular risk, PCSK9 is considered at present to be an important therapeutic target for combating hypercholesterolemia [15]. In addition, other factors that elevate circulating PCSK9 levels could be considered to be risk factors for coronary heart disease (CHD). A previous study by our group demonstrated that periodontitis, a chronic inflammatory disease induced by a group of periodontopathic bacteria, could elevate serum PCSK9 levels [9]. However, the underlying mechanisms by which periodontal infection affects PCSK9 levels and the subsequent alterations of the plasma lipid profile are not known.

It is well-known that infection and inflammation induce an acute phase response, which leads to multiple alterations in lipid and lipoprotein metabolism [10]. We have shown that chronic oral infection with *P. gingivalis* induced downregulation of HDL cholesterol by suppressing Liver X receptors and their target gene *Abca1* in C57BL/6 mice and ApoE-deficient C57BL/6.KOR-*Apoe*^*shl*^ (B6.Apoeshl) mice [16]. Furthermore, as in human periodontitis patients, the LDL cholesterol levels were elevated in *P. gingivalis*-infected B6.Apoeshl mice. However, the molecular basis for the elevation of LDL cholesterol was not analyzed in this experimental setting.

This study is the first to show that infection induces a significant increase in circulating PCSK9 levels and a concomitant decrease of LDLR levels in mice. Furthermore, there was a significant correlation found between LDLR gene expression in the liver and the serum levels of PCSK9. In this study, infection was induced via intraperitoneal, rather than oral, bacterial inoculation. Although oral infection can mimic human periodontal disease, and it is clear that oral infection with *P. gingivalis* does, in fact, affect *Pcsk9* gene expression in the liver (data not shown), subsequent change in lipid metabolism can be easily observed in peritoneal infection model.

*Pcsk9* gene expression was significantly upregulated in the liver of the *P. gingivalis*-infected mice. Previous studies have demonstrated that *Pcsk9* and *Ldlr* gene transcription is regulated by sterol regulatory element-binding proteins (SREBPs) [17,18]. Consistent with these studies, *Srebf2* expression was found to be significantly upregulated in the livers of the *P. gingivalis*-infected mice compared with the sham-infected mice. *Srebf2* expression is downregulated by cholesterol and upregulated by statin treatment. As total and LDL cholesterol were elevated in the *P. gingivalis*-infected mice, *Srebf2* is likely to be induced by this infection. Although the mechanisms by which infection and/or inflammation induce the upregulation of *Srebf2* are not clear, our study suggests that additional mechanisms related to infection induce hyperlipidemia during infection.

Recently, another transcription factor, known as hepatocyte nuclear factor-1α (HNF1α), was reported to be involved in the transcriptional regulation of the *Pcsk9* gene in mammalian HepG2 cells [19]. However, it has not been reported whether *Pcsk9* is activated by HNF1α in mice. Therefore, other mechanisms may regulate *Pcsk9* expression during infection; further studies will be needed to answer this question.

In contrast to the gene expression results, the level of mature PCSK9 protein in the liver was unchanged in the *P. gingivalis*-infected mice compared to the sham-infected mice, whereas the levels of PCSK9 proprotein were slightly reduced in the *P. gingivalis*-infected mice. Because we failed to observe the enhanced transcription of PCSK9 in other organ specimen such as liver, kidney, jejulium, ilenium, aolta, and spleen (data not shown), we speculate that secreted PCSK9 immediately infiltrate into circulation and result in the elevation of PCSK9 levels higher in the *P. gingivalis*-infected mice. Recently, it has become evident that domain interactions are important for the regulation of PCSK9 protein secretion [20]. However, it remains to be elucidated how infection affects PCSK9 protein secretion.

Similar to *Pcsk9*, *Ldlr* is upregulated in the livers of the *P. gingivalis*-infected mice compared with the sham-infected mice, although to a much lesser degree. Because LDLR expression is also positively regulated by SREBP2 [18], upregulation of *Ldlr* could be an effect of the infection. Contrary to what was observed regarding *Ldlr* gene expression, LDLR protein expression in the liver of the *P. gingivalis*-infected mice was significantly reduced compared with the sham-infected mice. Although the *Ldlr* gene expression levels in the *P. gingivalis*-infected mice were 2 times higher compared with the sham-infected mice, the expression of LDLR protein was decreased, possibly because PCSK9 serum levels were increased more than 20 fold in the infected mice.

In addition to PCSK9, LDLR expression is controlled by the LXR-Idol axis, in which LXR induces Idol, an E3 ubiquitin ligase that triggers LDLR degradation [5]. The importance of Idol in the regulation of LDLR expression is increasingly being recognized [21]. Therefore, the affect of *P. gingivalis* infection on *Idol* gene expression was analyzed. Although the expression of the *Idol* gene was significantly down-regulated in the *P. gingivalis*-infected mice, the difference in *Idol* gene expression between the infected and the sham-infected mice was smaller than what was observed for *Pcsk9*. In addition, the expression of *Lxrs* did not differ between the *P. gingivalis*-infected mice and the sham-infected mice. Therefore, it is possible that *P. gingivalis* infection does not affect LDLR expression by downregulating *Idol* in this experimental model.

Our study supports the previously described notion that bacterial infections alter the plasma lipid profile based on the novel finding that *P. gingivalis* infection elevates PCSK9 expression in the liver by increasing *Srebf2* expression through a currently unidentified mechanism. The effects of infection and inflammation on lipid and lipoprotein metabolism are complex, and contrasting results are often demonstrated between rodents and primates [10]. Our animal study model has potential limitations considering the characteristics of chronic inflammation in human periodontitis, because peritoneal infection in mice induces robust acute immune response. There may be other proteins that are involved in the regulation of PCSK9 by the mechanisms related to inflammation. Further studies will be required to gain insight into the role of PCSK9 in the dyslipidemia that accompanies infections and chronic inflammatory diseases, such as periodontitis.

## Conclusions

We found that *P. gingivalis* infection upregulates PCSK9 production via upregulation of *Srebf2*, independent of cholesterol levels resulting elevation of LDL cholesterol. Further studies are required to elucidate how infection regulates *Srebf2* expression and subsequently influences lipid metabolism.

## Methods

### Mice

All experiments were performed in accordance with the Regulations and Guidelines on Scientific and Ethical Care and Use of Laboratory Animals of the Science Council of Japan, enforced on June 1, 2006 and approved by the Institutional Animal Care and Use Committee at Niigata University (permit number 231–1). Six-week-old male C57BL/6 mice were obtained from Japan SLC, Inc. (Shizuoka, Japan). The mice were maintained under pathogen-free conditions and were fed regular chow and sterile water until they were infected.

### Bacterial cultures and infection

*P. gingivalis* strain W83 was cultured in modified Gifu anaerobic medium (GAM) broth (Nissui, Tokyo, Japan) in an anaerobic jar (Becton Dickinson Microbiology Systems, Cockeysville, MD) in the presence of an AnaeroPack™ (Mitsubishi Gas Chemical Co. Inc., Tokyo, Japan) for 48 hours at 37°C. Bacterial suspensions were prepared in Mg^2+^/Ca^2+^-free phosphate-buffered saline (PBS) using spectrophotometry to establish growth curves. The number of CFUs was standardized by measuring the optical density at 600 nm. The mice in the experimental groups were administered a single intraperitoneal (i.p.) inoculum of 10^9^ CFU in 0.2 ml of sterile phosphate-buffered saline (PBS), pH 7.2. Control mice were inoculated with PBS alone. Mice were euthanized 16 hrs after infection and then analyzed.

### Real-time PCR

Total RNA was isolated from the liver using TRIzol™ (Life Technologies, Carlsbad, CA) according to the manufacturer's instructions. Next, aliquots of RNA were reverse transcribed to produce cDNA using random primers (Takara Bio Inc., Shiga, Japan) and M-MLV reverse transcriptase (Life Technologies, Carlsbad, CA). Specific primers and probes for real-time PCR were purchased from Applied Biosystems (Foster City, CA). TaqMan Gene Expression Assays (Applied Biosystems) were performed in 20-μl reactions containing 900 nM primers and 250 nM probe using an ABI PRISM 7900HT Sequence Detection System (Applied Biosystems). The reaction conditions involved a 10-minute incubation at 95°C followed by 40 cycles of a two-step amplification procedure consisting of annealing/extension at 60°C for 1 minute and denaturation for 15 seconds at 95°C. ABI PRISM SDS 2.0 software (Applied Biosystems) was used to analyze the standards and quantify the data. The relative quantity of each mRNA sequence was normalized to the relative quantity of glyceraldehyde-3-phosphate dehydrogenase (GAPDH) mRNA.

### Western blotting

The livers of the mice were dissected into small pieces, and protein was extracted using T-PER Tissue Protein Extraction Reagent (Thermo Scientific, Rockford, IL) supplemented with a Halt Protease Inhibitor Cocktail Kit (Thermo Scientific) and Halt Phosphatase Inhibitor Cocktail (Thermo Scientific) according to the manufacturer’s instructions. Cell debris was pelleted by centrifugation at 10,000 *× g* for 5 minutes at 4°C. The protein concentration in the supernatant was determined using the Pierce BCA Protein Assay kit (Thermo Scientific) according to the manufacturer’s instructions.

Thirty micrograms of each sample were solubilized in SDS sample buffer, separated by SDS-PAGE, and transferred to a polyvinylidene difluoride membrane (Immobilon-P; Millipore Co., Bedford, MA). The samples were subsequently subjected to western blotting with rabbit anti-mouse PCSK9 (Abcam, Cambridge, UK) or rabbit anti-mouse LDLR (Abcam) primary antibodies followed by ECL™ Peroxidase labeled anti-rabbit secondary antibodies (GE Healthcare, Buckinghamshire, UK). The blots were developed with ECL using Lumi Vision PRO 400EX (Aisin, Aichi, Japan). The membranes were then washed 3 times with wash buffer (Tris-buffered saline containing 0.1% Tween-20 and 0.5% BSA) and reprobed with goat anti-mouse GAPDH antibodies (Santa Cruz Biotechnology, Santa Cruz, CA), as described above. Horseradish peroxidase-labeled donkey anti-goat IgG (Santa Cruz) was used as secondary antibody for reprobing. The intensity of the signal was quantified with Scion Image 4.02 computer software. The intensity of each protein band was normalized to the intensity of GAPDH.

### Serum PCSK9, SAA, and lipoprotein levels

SAA and PCSK9 were measured using commercially available ELISA kits (Tridelta Development Ltd., Kildare, UK and R & D Systems, Minneapolis, MN, respectively) in serum collected prior to euthanasia. Serum cholesterol and triglyceride profiles were analyzed at Skylight Biotech Inc. (Akita, Japan).

### Statistical analyses

The differences in the examined gene expression and biochemical parameters between the infected and control mice were analyzed using the Mann–Whitney U-test. Linear correlations were obtained using Spearman’s rank correlation coefficient analysis. Unpaired t-tests were used for densitometric analysis. The statistical analyses were performed using standard statistical software (GraphPad Prism, GraphPad Software Inc., La Jolla, CA and StatView J-5.0 application program, SAS Institute Inc., Cary, NC). *P* < 0.05 was considered to be statistically significant.

## Abbreviations

PCSK9: Proprotein convertase subtilisin/kexin type 9; LDL: Low-density lipoproteins; HDL: High-density lipoproteins; LDLR: LDL receptor; LXR: Liver X receptor; Idol: Inducible degrader of the LDLR; Srebf2: Sterol regulatory element binding transcription factor2; SREBP: Sterol regulatory element binding protein; LPS: Lipopolysaccharide; B6.Apoeshl: C57BL/6.KOR-*Apoe*^*shl*^; HNF1α: Hepatocyte nuclear factor-1.

## Competing interests

The authors declare that they have no competing interests.

## Authors’ contributions

KT, TN, and KY designed the study; HM, SM, YA-N, HD and TM performed the experiments; KT, TN and KY wrote the paper. All authors read and approved the final manuscript.
